# Letter to Editor: Development of proliferative verrucous leukoplakia (PVL)in oral lichen planus: Is it not a clinical spectrum of PVL?

**DOI:** 10.4317/medoral.21605

**Published:** 2017-02-04

**Authors:** Diego-Tetzner Fernandes, Alan-Roger Santos-Silva, Pablo-Agustin Vargas, Márcio-Ajudarte Lopes

**Affiliations:** 1 DDS, PhD student. Oral Diagnosis Department, Piracicaba Dental School, University of Campinas – UNICAMP, Piracicaba, São Paulo, Brazil; 2 DDS, PhD. Oral Diagnosis Department, Piracicaba Dental School, University of Campinas – UNICAMP, Piracicaba, São Paulo, Brazil

Dear Editor: In their recent publication, Garcia-Pola *et al.* (1) (2016) suggested the development of proliferative verrucous leukoplakia (PVL) in oral lichen planus (OLP). It was described as a preliminary study, where this association supposedly occurred. They presented a retrospective analysis with a long-term follow-up, showing an interesting relationship between these lesions. It is important to emphasize that the clinical profile of patients with PVL is well recognized in the literature, mainly affecting non-smoking and non-drinking elderly women, older than 60 years (2-4). We have diagnosed in our institution patients that initially present oral lichenoid lesions throughout the oral musosae. However, some years after close follow-up, some lichenoid areas became clinically verrucous leukoplakia leading to the diagnosis of PVL (5). Interestingly, the histopathological analysis of these initial lichenoid lesions often was not confirmed as lichenoid reaction or even lichen planus. Over time, as these lesions changed clinically, other biopsies were performed and the histopathological aspects were compatible with the clinical hypothesis presenting hyperkeratosis and acanthosis and variable degrees of epithelial dysplasia. In addition, one patient with this profile presented initially lichenoid areas in both buccal mucosa and lateral border of the tongue bilaterally and fifteen months later developed squamous cell carcinoma on the left lateral border of the tongue. This case was recently published by our group (5) where it was suggested that, in some cases, the initial clinical manifestation of PVL may mimic OLP or oral lichenoid reaction. In our opinion, figures 1,2,3 and 4 of the paper of Garcia-Pola’s group did not represent a true oral lichen planus. In addition, histopathological figures could be provided to better illustrate the diagnosis of OLP. Therefore, differently from what was proposed by Garcia-Pola *et al.* (1) we believe that their cases represent an under-recognized spectrum of PVL, which is characterized by a lichenoid pattern and could lead to misdiagnosis. Sincerely,
